# Arteria Lusoria: An Anomalous Finding during Right Transradial Coronary Intervention

**DOI:** 10.1155/2016/8079856

**Published:** 2016-07-05

**Authors:** David Allen, Hilary Bews, Minh Vo, Malek Kass, Davinder S. Jassal, Amir Ravandi

**Affiliations:** ^1^Section of Cardiology, Department of Internal Medicine, Max Rady College of Medicine, Rady Faculty of Health Sciences, University of Manitoba, Winnipeg, MB, Canada; ^2^Department of Radiology, Max Rady College of Medicine, Rady Faculty of Health Sciences, University of Manitoba, Winnipeg, MB, Canada; ^3^Institute of Cardiovascular Sciences, St. Boniface Albrechtsen Research Centre, University of Manitoba, Winnipeg, MB, Canada

## Abstract

Arteria Lusoria or aberrant right subclavian artery (ARSA) is present in 0.6–1.4% of individuals. It typically remains clinically silent and is often discovered during angiographic procedures. The presence of ARSA can make a right transradial approach for coronary angiography and angioplasty technically more difficult. With the use of catheter support, we describe two cases in which a right transradial approach for catheterization was successful in the setting of ARSA. As such, the presence of ARSA does not warrant abandoning a transradial approach for coronary angiography and angioplasty.

## 1. Introduction

Arteria Lusoria or aberrant right subclavian artery (ARSA) is the most common congenital arch anomaly in which the right subclavian artery originates from the descending aorta, distal to the left subclavian at the ductus arteriosus [1]. On its course towards the right arm, the aberrant vessel travels retrotracheal and retroesophageal [1]. The prevalence of ARSA ranges from 0.6 to 1.4% [1]. The prevalence of ARSA rises exponentially to 26–34% in individuals with Down syndrome and other chromosomal defects [1].

ARSA remains clinically silent in the majority of cases. In approximately 5% of individuals, ARSA is associated with an aberrant umbilical vein, tricuspid atresia, or tetralogy of Fallot [1]. Rarely, ARSA can accompany Kommerell's diverticulum, an aneurysm of the descending aorta at the origin of the ARSA [2]. This can present clinically as dysphagia, dyspnea, or subclavian steal syndrome, often requiring surgical intervention [2].

Despite reports of complications on right radial approach for angiography, we report two cases demonstrating the success and safety of this approach.

## 2. Case  1

**Figure 1:**
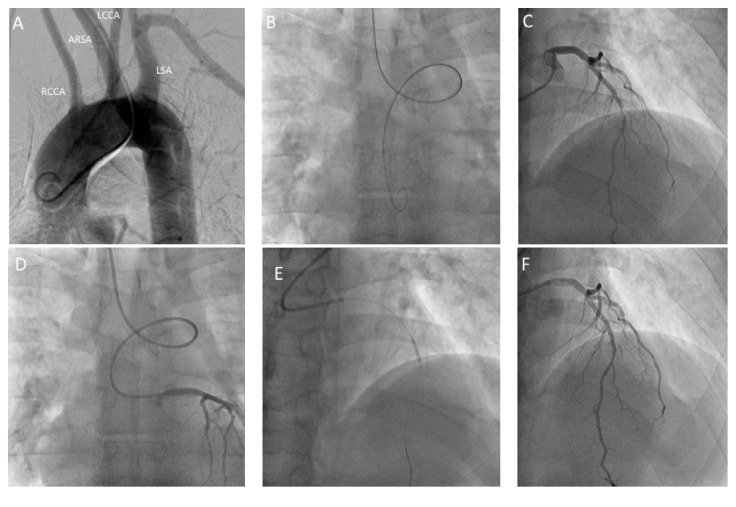
Angiographic images from Case  1: (A) aortogram of the ascending, transverse, and descending aorta demonstrating the ARSA; (B) anterior-posterior projection demonstrating the characteristic loop of ARSA with the guidewire in the descending aorta; (C) left anterior oblique cranial projection with Judkins left catheter engaging the left main demonstrating an 80% mid LAD coronary artery lesion; (D) the characteristic loop of ARSA; (E) ballooning of the LAD lesion; (F) final result. ARSA, aberrant right subclavian artery; LAD, left anterior descending; RCCA, right common carotid artery; LCCA, left common carotid; LSA, left subclavian artery.

A 42-year-old man with a past history of hypertension and hyperlipidemia, presented to hospital with acute onset of retrosternal chest discomfort. He had an elevation of his cardiac biomarkers and was diagnosed with non-ST elevation myocardial infarction (NSTEMI). During coronary angiography, right radial access with a 6 Fr. Side arm sheath was obtained. The advancement of any catheters into the ascending aorta from the radial approach was very difficult due to tortuous angulation at the junction between the ascending aorta and the right innominate artery. With the help of a 0.035′′ Glidewire (Terumo Interventional Systems, Somerset, NJ), a pigtail was advanced into the ascending aorta and an aortic root angiography was performed. This confirmed the presence of ARSA (Figure 1). For catheter exchanges, a 260 cm 0.038′′ J curved wire (Cordis, Hialeah, FL) was used. A JL3.5 diagnostic catheter (Cordis, Hialeah, FL) was used for left coronary angiography and a JR4.0 diagnostic catheter (Cordis, Hialeah, FL) was used for right coronary angiography. For the PCI procedure, a 6 Fr. XB 3.5 guiding catheter (Cordis, Hialeah, FL) was used to allow adequate support during the LAD intervention.

## 3. Case  2

**Figure 2:**
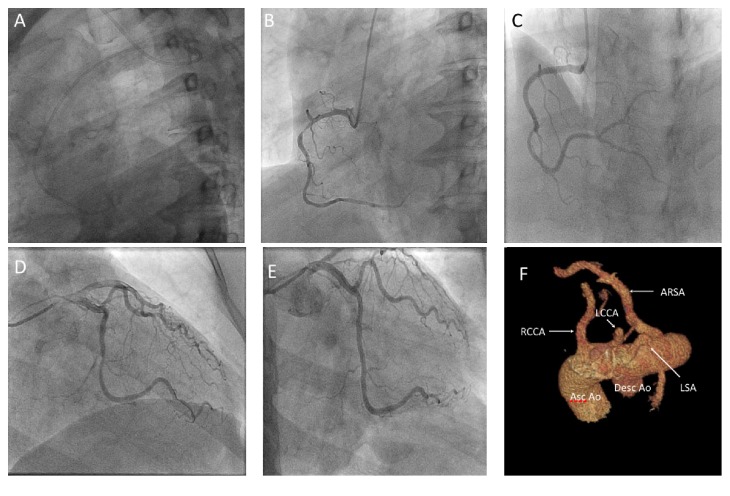
Angiographic images from Case  2: (A) lateral projection showing characteristic loop of ARSA; (B) left anterior oblique view of right coronary artery showing a 90% proximal lesion; (C) with subsequent stenting; (D) right anterior oblique caudal view of left coronaries with a 70% circumflex, 90% OM1, and 70% OM3; (E) final result; (F) A 3D volume rendered imaged from a contrast-enhanced CT angiographic dataset demonstrating vessels arising from the arch in the following order: RCCA, LCCA, LSA, and ARSA. ARSA, aberrant right subclavian artery; OM, obtuse marginal; RCCA, right common carotid artery; LCCA, left common carotid; LSA, left subclavian artery; Asc Ao, ascending aorta; Desc Ao, descending aorta.

A 51-year-old woman presented for angiography following a NSTEMI. A right radial approach with a 6 Fr. Sheath was selected. Multiple guidewires consistently engaged the descending aorta. A Judkins right 4.0 catheter (Cordis, Hialeah, FL) was placed at the right subclavian artery ostium. With gentle clockwise motion and simultaneous advancement of the 0.038′′ guidewire, the ascending aorta was successfully engaged (Figure 2). As with Case  1, catheter exchanges were done with a 260 cm 0.038′′ J curved wire (Cordis, Hialeah, FL). Angioplasty was performed using a 6 Fr. XB 4.0 guiding catheter (Cordis, Hialeah, FL). A subsequent computed tomography of the chest confirmed the diagnosis of ARSA.

## 4. Discussion

In the vast majority of patients, as in the two cases illustrated, ARSA is clinically silent until right radial coronary angiography is entertained. With the increasing use of a transradial approach for coronary angiography as a result of the lower risk of access site related complications, ARSA will be encountered more frequently [3]. ARSA can be confirmed by aortography and should be suspected when catheterization of the ascending aorta proves difficult and the catheter favors entry into the descending aorta. Due to the increased anatomical complexity, ARSA may increase number of catheters used and prolong angiography time, especially if previously unrecognized [3].

Previous studies have described low procedural success rates of 6/10 during catheterization in the setting of ARSA, due to increased technical demand [4]. We have demonstrated with the two current cases that catheter support and rotation can facilitate successful, nontraumatic entry into the ascending aorta [3]. In addition, a transradial approach in the setting of ARSA appears safe, with no significant difference in dissection rate between anatomically normal and variant subclavian arteries [4]. As in all cases in which complex percutaneous coronary intervention is required, specific attention should be placed on appropriate guide support. In the absence of this, consideration towards gaining access from another approach would be warranted.

## 5. Conclusion

While right radial angiography and angioplasty are technically more demanding in the setting of ARSA, it does not necessitate abandoning the right radial approach [5].
